# Cost Analysis of Individualized Parenteral Nutrition Bags in a Saudi Tertiary-Care Hospital: A Retrospective Cohort Study and Implications for Cost-Effective Clinical Practice

**DOI:** 10.3390/healthcare14050658

**Published:** 2026-03-05

**Authors:** Nora Albanyan, Mrayam Almuzayen, Aljawharah BinRokan, Sarah Alotaibi, Joud Alotaibi, Razan Orfali, Michael Freudiger

**Affiliations:** 1 Clinical Pharmacy Department, Riyadh Second Health Cluster, Riyadh 12231, Saudi Arabia; nalbanyan@gmail.com (N.A.);; 2 Research Center, Riyadh Second Health Cluster, Riyadh 12231, Saudi Arabia; 3 Department of Pharmacology, College of Medicine, Imam Mohammad Ibn Saud Islamic University (IMSIU), Riyadh 13317, Saudi Arabia; 4 Valley Children’s Healthcare Pharmacy, Madera, CA 93636, USA; 5 Saint Agnes Medical Center Pharmacy, Fresno, CA 93720, USA

**Keywords:** parenteral nutrition, cost analysis, health economics, pharmaceutical care, clinical pharmacy, cost drivers, Saudi Arabia, tertiary-care hospital

## Abstract

**Background:** Parenteral nutrition (PN) is a life-sustaining therapy essential for patients who are unable to meet their nutritional needs enterally. However, individualized PN formulations impose substantial economic burdens on healthcare systems. This study aims to quantify the cost of individualized PN bags across different patient populations and identify key cost drivers to inform cost-effective clinical practice and policy development. **Methods:** A retrospective cohort study was conducted at King Fahad Medical City, Riyadh, Saudi Arabia, analyzing 900 unique patient-specific PN orders between February 2023 and August 2023. Patients were stratified into three groups: adults (≥18 years), pediatrics (1 month to 17 years), and neonates (<1 month), with 300 unique patients per group. The cost assessment included macronutrients, micronutrients, consumables, equipment, and personnel time, all measured using a standardized work sampling methodology. Descriptive statistics characterized demographic and clinical profiles. One-way ANOVA was used to compare costs across groups, and multivariate linear regression identified significant cost predictors, with log-transformation applied to address the skewness in the cost data. **Results:** Mean cost per PN bag varied significantly among patient groups (ANOVA, *p* < 0.001): adults 517.1 ± 274 SAR, pediatrics 383.2 ± 86.75 SAR, and neonates 243.14 ± 98 SAR. We found that PN volume, lipid dose, and the number of additives were the primary modifiable drivers of PN cost. Multivariate regression analysis identified PN volume (β = 0.182, *p* < 0.001), lipid dose (β = 0.145, *p* = 0.002), and number of additives (β = 0.098, *p* = 0.028) as significant predictors of cost, explaining 91.2% of the cost variance (R^2^ = 0.912). Consumables contributed 18–22% of total costs across groups. Pediatric patients demonstrated markedly longer therapy duration (median 98 days, IQR 65–142) compared to adults (median 18 days, IQR 8–35) and neonates (median 24 days, IQR 12–42). **Conclusions:** This study provides the first stratified, real-world cost benchmarks for individualized PN in a Saudi tertiary-care setting and quantifies actionable cost drivers. Actionable implications include standardizing stable-patient procedures, implementing pharmacist-led appropriateness screening, and earlier transition to enteral nutrition to reduce costs while maintaining quality of care. Future research should evaluate the cost-effectiveness of standardized versus individualized formulations and investigate the relationship between cost variations and clinical outcomes.

## 1. Introduction

Parenteral nutrition (PN) represents a critical life-sustaining intervention that delivers essential nutrients directly into the bloodstream for patients unable to obtain adequate nutrition through oral or enteral routes [1,2,3]. PN serves as a vital therapeutic option for individuals undergoing major surgery, managing severe gastrointestinal dysfunction, or experiencing critical illness. A typical PN formulation comprises complex admixtures of macronutrients (amino acids, carbohydrates, and lipids) and micronutrients (electrolytes, trace elements, and vitamins) [4]. The preparation process demands meticulous multi-step procedures encompassing prescription, order review, compounding, dispensing, administration, and continuous monitoring [4,5], making PN one of the most complex prescriptions routinely managed in hospital settings [6].

Recent guidelines from the American Society for Parenteral and Enteral Nutrition (ASPEN) emphasize patient safety, prevention of nutrition-related complications, and strategies to mitigate unnecessary economic burdens [7,8,9,10]. These guidelines advocate for evidence-based standardization as a critical strategy to reduce prescribing errors, enhance safety, and control costs. Pharmacists have evolved from traditional compounding roles to integral members of multidisciplinary healthcare teams, contributing specialized clinical expertise in nutrition support therapy [11]. This evolution aligns with the pharmaceutical care model, wherein pharmacists actively manage medication therapy to improve patient outcomes and enhance quality of life [12,13,14]. Their contributions extend to appropriateness screening, enteral transition planning, and therapy duration optimization, yielding significant cost savings and improved resource allocation [13,15].

### 1.1. Research Gap and Study Rationale

Economic evaluations of PN services have revealed substantial cost variability across healthcare settings. A multicenter European study identified significant differences in average PN bag costs, with staff time accounting for a considerable proportion of overall expenses [16]. Hospital-prepared PN bags reported an average cost of €37.64 per bag, with raw materials accounting for the majority of the expense [17]. Three-compartment bag systems reduced daily costs by €9.36 per patient compared to multi-bottle systems over 10-day regimens [18]. Petrelli et al. (2004) compared hospital-prepared TPN bags with commercially prepared bags after supplementation and reported that the total costs were broadly comparable, with supplemented commercial preparations being almost equal in cost to hospital preparations with similar nutritional value and safety [19].

Despite international evidence on PN costs, a significant gap remains in real-world cost data from Middle Eastern healthcare settings, particularly regarding cost stratification across different patient populations. Local and international guidelines recommend standardized PN for clinically stable patients [20,21,22], yet the financial impact of individualized PN formulations in Saudi tertiary-care settings remains poorly characterized. Furthermore, the existing literature lacks a comprehensive identification of modifiable cost drivers to inform targeted cost-containment strategies while maintaining clinical appropriateness. This study addresses these gaps by providing the first comprehensive, stratified cost analysis of individualized PN therapy in Saudi Arabia, employing rigorous cost accounting methodology that separates direct ingredient costs, consumable costs, and measured personnel time to identify specific, modifiable cost drivers for optimizing clinical practice.

### 1.2. Research Question and Objectives

Primary Research Question: What is the cost structure of individualized parenteral nutrition therapy across different patient populations in a Saudi tertiary-care hospital, and what are the key modifiable cost drivers?

More specifically, the study aims to:

1. Quantify and compare the cost per PN bag across adult, pediatric, and neonatal populations;
2. Identify and rank significant predictors of PN cost variation;
3. Characterize the contribution of different cost components (nutrients, consumables, and personnel) to total PN costs

These objectives directly address the research gap by providing baseline cost benchmarks essential for future cost-effectiveness evaluations comparing individualized PN with standardized formulations or multi-chamber bag systems, and for quantifying the economic impact of pharmacist-led interventions.

### 1.3. Study Contribution

This study makes several distinct contributions to the fields of health economics and clinical pharmacy. First, it provides a comprehensive, stratified cost analysis of individualized PN therapy in a Saudi tertiary-care setting, filling a significant gap in both geography and the healthcare system. Second, it employs a rigorous cost accounting methodology that separates direct ingredient costs, consumable costs, and measured personnel time, providing granular data on cost components. Third, by identifying specific, modifiable cost drivers through multivariate analysis, this research provides actionable insights for optimizing clinical practice. Finally, it establishes baseline cost benchmarks essential for future cost-effectiveness evaluations comparing individualized PN with standardized formulations or multi-chamber bag systems, and for quantifying the economic impact of pharmacist-led interventions.

## 2. Literature Review

### 2.1. Economic Burden of Parenteral Nutrition

Parenteral nutrition therapy represents a substantial cost category in hospital pharmacy budgets globally. The economic burden extends beyond direct ingredient costs to encompass preparation time, consumables, monitoring, and potential complications. Correia et al. [23] examined the economic burden of hospital malnutrition in Latin America, demonstrating that supplemental PN in critically ill patients, while costly, could be cost-effective when used to prevent malnutrition-related complications. Sadique et al. [24] conducted a cost-effectiveness analysis comparing early nutritional support via parenteral versus enteral routes for critically ill adults, finding that enteral nutrition was generally more cost-effective but that PN remained necessary for specific patient subgroups.

Seres, Valcarcel, and Guillaume (2013) reported that malnutrition and suboptimal nutrition support are strongly associated with poorer clinical outcomes and increased healthcare utilization, emphasizing that inadequate nutritional management generates downstream economic consequences [25].

In critically ill populations, It was demonstrated that early and adequate nutrition therapy, although initially resource-intensive, was associated with reduced overall hospital costs through shorter length of stay and fewer complications [18]. Similarly, Pradelli et al. (2018) conducted comprehensive cost-effectiveness modeling of supplemental PN (SPN) in intensive care unit (ICU) patients and reported that SPN administered on days 4–8 was associated with a significant reduction in cumulative energy deficit compared to enteral nutrition (EN) alone [26]. This decrease in energy deficit correlated with a 10% reduction in the risk of nosocomial infection for every 1000 kcal decrease.

The economic burden of PN extends beyond formulation costs to include therapy-related complications. Gamsjäger et al. (2009) found that individualized parenteral nutrition admixtures were significantly more costly per patient per day than standard commercial solutions in critically ill pediatric patients, highlighting the economic impact of PN formulation choices on overall healthcare costs [21]. Matlow et al. (1999) conducted a randomized trial comparing 72-h versus 24-h intravenous tubing changes in neonates receiving lipid therapy and demonstrated that less frequent tubing changes did not increase catheter-related infection rates, suggesting an opportunity to reduce costs and resource use associated with IV care [22].

### 2.2. Cost Variability Across Patient Populations

Pediatric and neonatal populations present unique challenges in PN therapy, with distinct cost structures compared to those of adults. Jacobs et al. reviewed early supplemental parenteral nutrition in critically ill children, highlighting both clinical outcomes and economic considerations [27]. Protheroe discussed long-term parenteral nutrition in pediatrics, noting that prolonged therapy duration substantially increases cumulative costs [23]. Walter et al.’s multicentre European study specifically examined neonatal and pediatric PN costs, identifying significant variability across countries and highlighting that personnel time constituted a major cost component [17].

Recent advances in neonatal PN have further altered cost structures. Embleton noted that newer lipid emulsions, including mixed and fish-oil-containing formulations, improve clinical outcomes but increase ingredient costs by approximately 30–40% [24,27]. Protheroe et al. (2019) demonstrated that despite higher upfront costs, optimized lipid provision in preterm infants may reduce long-term neurodevelopmental complications and associated healthcare expenditures [28].

Beyond the neonatal period, pediatric patients receiving long-term PN for chronic intestinal failure generate substantially higher cumulative costs than acute-care populations. Diamanti et al. (2014) analyzed pediatric home PN and found that although daily PN costs were lower in-home settings than in hospitals, extensive caregiver training, monitoring, and complication management created substantial indirect and hidden costs [29].

Kochevar et al. (2007) stated that parenteral nutrition standardization should be implemented as a comprehensive process—including standardized formulations as well as ordering, labeling, screening, compounding, and administration—to improve patient safety, reduce procedural incidents, and enhance resource efficiency [30].

### 2.3. Cost Components and Drivers

The literature identifies several key cost components in PN therapy. Menne et al. (2008) provided a detailed cost analysis comparing three-compartment bag systems with multi-bottle systems, demonstrating that simplified preparation methods could reduce both direct costs and personnel time [18]. Petrelli et al. emphasized that raw materials (amino acids, lipids, dextrose) typically constitute 60–70% of direct costs, with consumables and preparation supplies contributing 15–25% [19]. Hidden costs, including waste from partially used ingredient vials, have been estimated at 8–10% of ingredient costs according to National Institute for Health and Care Excellence guidelines [31].

Lipid emulsions represent a particularly important cost driver. Driscoll (2006) reported that newer fish-oil-based and mixed lipid emulsions cost two to three times more than traditional soybean-based emulsions [32]. Nevertheless, Mayer et al. (2020) argued that improved clinical outcomes associated with omega-3-containing lipids may offset higher acquisition costs by reducing inflammatory complications [33].

Infrastructure and equipment costs are frequently overlooked in cost analyses. Menne et al. (2008) noted that laminar airflow hoods required for safe PN compounding represent capital investments of €30,000–€60,000, with annual maintenance costs of €5000–€8000, contributing significantly to per-bag costs in lower-volume facilities [18].

### 2.4. Role of Clinical Pharmacists in Cost Optimization

Pharmacist involvement in PN therapy has consistently demonstrated both clinical and economic benefits. Mutchie et al. [16] conducted an early study demonstrating that pharmacist monitoring of parenteral nutrition was both clinically and cost-effective. More recently, Katoue [12] comprehensively reviewed the role of pharmacists in parenteral nutrition support, identifying multiple intervention points including appropriateness screening, formulation optimization, and enteral transition facilitation. Riordan et al.’s [34] systematic review of pharmacist-led interventions in optimizing prescribing demonstrated significant cost savings across multiple therapeutic areas, with applicability to nutrition support.

The American Society for Parenteral and Enteral Nutrition (ASPEN) has formally recognized the central role of pharmacists in nutrition support [7]. A.S.P.E.N. Parenteral Nutrition Safety Consensus Recommendations emphasize that pharmacist involvement is essential in PN safety, including order review/verification, assessment of compatibility and stability, and ongoing monitoring as part of the nutrition support process [35]. Pharmacist-led PN review generates multiple clinically relevant interventions per patient, reflecting the high opportunity for error prevention [36].

### 2.5. Standardization and Protocol Development

Standardization of PN formulations for clinically stable patients has emerged as a key cost-containment strategy. Kochevar et al. outlined ASPEN’s position on PN standardization, emphasizing safety and efficiency benefits [30]. Crews et al. demonstrated that standardization combined with electronic ordering systems reduced errors and improved workflow efficiency [37]. Mourkogianni et al. [38] assessed and optimized pediatric PN preparation processes, showing that systematic process improvements could reduce both costs and preparation time.

The safety benefits of standardization have been well documented. Ayers et al. (2014) synthesized safety recommendations from international PN consensus statements, emphasizing that standardization reduces complexity-related errors [35]. A recent review done in Saudi Arabia showed that a structured approach to integrating automated compounding devices with Epic, incorporating standardized data exchange protocols and interoperability frameworks.

Comparative effectiveness research has examined standardized versus individualized approaches. Ridley et al. (2018) found that an individually titrated supplemental parenteral nutrition strategy applied over the first 7 days in critically ill adults significantly increased energy delivery closer to estimated requirements compared with usual care (mean total calories delivered 1712 vs. 1130, *p* < 0.0001), as well as a higher percentage of estimated energy requirement achieved (83% vs. 53%, *p* < 0.0001) [39].

However, successful standardization requires careful implementation. Boullata et al. (2017) outlined best practices for PN standardization, emphasizing the need for multidisciplinary input, regular protocol review, and maintenance of individualization capacity for complex patients [7].

### 2.6. Theoretical Framework and Hypotheses

Based on the literature review, this study employs a cost-driver analysis framework, wherein total PN cost is conceptualized as a function of the following: (1) patient characteristics (age group, weight, and clinical acuity), (2) formulation complexity (volume, lipid content, and number of additives), and (3) operational factors (preparation time, consumables).

**Figure 1:**
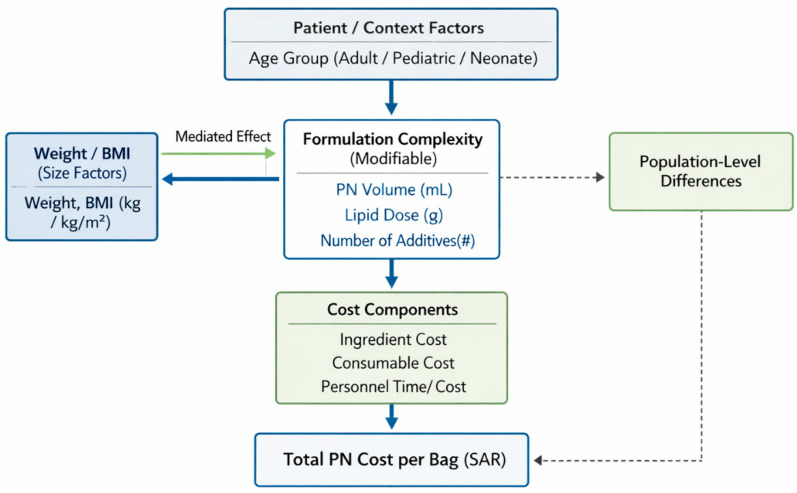
**Conceptual framework.** Total PN cost per bag is modeled as a function of formulation complexity (PN volume, lipid dose, and number of additives), which drives ingredient, consumable, and personnel costs. Age group captures population-level differences that may persist beyond formulation characteristics. Weight/BMI effects are hypothesized to be mediated through PN volume.

This framework aligns with established health economics methodologies for cost-driver analysis. Drummond et al. describe cost component analysis and the identification of cost drivers as core elements of economic evaluation in healthcare [40]. Muennig and Bounthavong outline regression-based and modeling approaches for identifying predictors and drivers of healthcare costs [41]. The framework enables identification of specific intervention points for cost optimization while maintaining clinical appropriateness (Figure 1).

**Research Hypotheses:**

**H1.**  *PN cost per bag differs significantly across patient age groups (adults, pediatrics, and neonates).*

**H2.**  *PN volume is a significant positive predictor of PN cost.*

**H3.**  *Lipid dose is a significant positive predictor of PN cost.*

**H4.**  *The number of additives is a significant positive predictor of PN cost.*

**H5.**  *Patient weight and BMI are not independent predictors of PN cost after controlling for PN volume.*

## 3. Methods

### 3.1. Study Design and Setting

This retrospective cohort study was conducted at King Fahad Medical City (KFMC), a 1200-bed tertiary-care hospital in Riyadh, Saudi Arabia. KFMC serves as a major referral center providing comprehensive medical services, including intensive care, neonatal care, complex surgery, and oncology. The hospital’s pharmacy department operates a centralized sterile compounding facility that prepares all individualized PN formulations for inpatient and outpatient services.

### 3.2. Study Population and Sampling

The study included all patients across all age groups who received individualized (hospital-compounded) parenteral nutrition between 1 February 2023 and 31 August 2023. Patients were stratified into three age categories based on established nutritional and physiologic differences: neonates: <1 month of age, pediatrics: 1 month to 17 years, and adults: ≥18 years

**Sampling Strategy:** To ensure independence of observations and avoid clustering bias, we selected one PN order per patient at random using a computer-generated random number sequence. This approach ensured that each PN order in the analysis represented a unique patient, eliminating potential clustering effects from multiple orders from the same individual. From eligible patients in each age group, we randomly selected 300 unique patients per group, yielding a total sample of 900 unique PN orders from 900 distinct patients.

**Inclusion Criteria:**

- Patients receiving individualized (compounded) PN during the study period;
- Complete medical and pharmacy compounding records available;
- PN orders with complete cost documentation.

**Exclusion Criteria:** None applied; all patients meeting the inclusion criteria were eligible for random selection.

**Sample Size Justification:** The sample size of 300 patients per group was determined based on: (1) detecting a minimum clinically meaningful cost difference of 50 SAR between groups with 80% power at α = 0.05, (2) anticipated cost standard deviations from pilot data, and (3) pragmatic feasibility considerations for comprehensive cost data extraction.

### 3.3. Data Collection

Data were systematically extracted from two primary sources: (1) electronic medical records (Cerner Millennium), and (2) pharmacy compounding records (pharmacy information system). A standardized data extraction form (Annex 1) was developed and pilot-tested on 20 cases before full implementation.

Data reliability was ensured through: (1) double-entry verification for 10% of records with 98.5% concordance, (2) automated range checks for biologically implausible values, and (3) systematic review of missing data patterns. Missing data (<2% for all variables) were handled through complete case analysis, given the low missingness rate.

**Cost Variables:** All costs were obtained from KFMC’s internal procurement database (SAP system) and pharmacy cost accounting records, reflecting actual acquisition costs as of Q2–Q3 2023. Costs are reported in Saudi Riyals (SAR). For literature comparison purposes, costs reported in euros were converted to SAR using the European Central Bank exchange rate for June 2023 (1 EUR = 4.08 SAR) and adjusted for inflation to 2023 values using the Consumer Price Index.

### 3.4. Cost Calculation Methodology

The primary outcome was cost per PN bag per day, calculated as the sum of three major cost components:

#### 3.4.1. Nutritional Ingredient Costs

Costs for each ingredient were calculated based on actual volume or weight used:

- Amino acids (per gram);
- Dextrose (per gram);
- Lipid emulsions (per mL);
- Electrolytes: sodium, potassium, calcium, magnesium, and phosphate (per mEq or mmol);
- Trace elements (per dose);
- Multivitamins (per dose);
- Specialized additives (insulin, heparin, and H2 antagonists, as applicable).

**Waste Consideration:** This analysis includes only ingredients incorporated into dispensed PN bags. Residual waste from partially used vials, estimated at 8–10% in the literature [34], was not quantified in this study and represents a limitation discussed in Section 5.

#### 3.4.2. Consumable Costs

All consumables required for PN preparation were itemized and costed

- PN bags (various sizes);
- Transfer sets and tubing;
- Syringes (1, 3, 5, 10, 20, and 50 mL);
- Needles (19G standard and filter needles);
- Sterile gloves;
- Personal protective equipment (PPE): shoe covers, head covers, face masks, and surgical gowns;
- Cleaning supplies: sterile water for injection (SWFI), antiseptics, and gauze.

**Consumable Allocation:** Fixed daily consumables (PPE and cleaning supplies) were allocated proportionally based on the number of PN bags prepared per compounding session. Variable consumables were assigned per PN bag based on standard operating procedures.

#### 3.4.3. Personnel Costs

**Time Measurement Methodology:** Personnel time was measured using a standardized work sampling methodology. Two trained observers conducted time-and-motion studies over 15 separate compounding sessions during the study period, and recording time was spent on:

- Order review and verification (pharmacist);
- Calculation and documentation (pharmacist);
- Ingredient preparation and compounding (pharmacy technician);
- Final verification and quality check (pharmacist);
- Labeling and dispensing (pharmacy technician).

Each PN bag was observed from receipt of the order to final dispensing. To minimize observer-related bias, observers were trained on standardized operational definitions, used the same structured observation form, and conducted pilot observations prior to data collection. Mean time per PN bag was calculated separately for each age group, as neonatal and pediatric formulations typically require more calculations and smaller-volume manipulations. Personnel costs were calculated using KFMC salary scales, including benefits:

- Clinical pharmacist: 180 SAR/h;
- Pharmacy technician: 90 SAR/h.

**Personnel Time Allocation:** For each PN bag, personnel cost = (pharmacist time × pharmacist hourly rate) + (technician time × technician hourly rate).

**Standard Operating Times Established:**

- Adult PN: Pharmacist 25 min, Technician 35 min;
- Pediatric PN: Pharmacist 30 min, Technician 40 min;
- Neonatal PN: Pharmacist 35 min, Technician 45 min.

### 3.5. Variables and Operational Definitions

**Dependent Variable:**

- Total cost per PN bag (SAR): Sum of ingredient, consumable, and personnel costs.

**Independent Variables:**

- Age group (categorical): neonates, pediatrics, adults;
- PN volume (continuous, mL);
- Lipid dose (continuous, g);
- Number of additives (continuous count);
- Patient weight (continuous, kg);
- Body mass index (continuous, kg/m^2^; adults and pediatrics only);
- Therapy duration (continuous, days).

**Control Variables:**

- Patient location (ICU vs. ward vs. outpatient);
- Primary indication for PN.

### 3.6. Statistical Analysis

All statistical analyses were performed using SPSS version 28.0 (IBM Corp., Armonk, NY, USA) and R version 4.3.0 (R Foundation for Statistical Computing).

#### 3.6.1. Descriptive Statistics

Patient demographics and clinical characteristics were summarized using appropriate descriptive statistics:

- Continuous variables: mean ± standard deviation (SD) and median with interquartile range (IQR), particularly for highly skewed variables (therapy duration);
- Categorical variables: frequencies and percentages.

Normality of the distribution was assessed using the Shapiro–Wilk tests and visual inspection of Q-Q plots.

#### 3.6.2. Comparative Analysis

One-way analysis of variance (ANOVA) was used to test H1 by comparing mean PN costs across the three age groups. Given the skewed distribution of cost data (Shapiro–Wilk test, *p* < 0.001), we conducted sensitivity analysis using the Kruskal–Wallis H test (non-parametric alternative) and ANOVA on log-transformed costs.

#### 3.6.3. Multivariate Regression Analysis

**Model Specification:** Multivariate linear regression was performed to test hypotheses H2 (PN volume as a predictor), H3 (lipid dose as a predictor), H4 (additives as a predictor), and H5 (weight/BMI non-significance after controlling for volume). Due to the right-skewed distribution typical of cost data, the dependent variable (total PN cost) was natural log-transformed to satisfy regression assumptions of normality and homoscedasticity.

**Model Equation:** ln(Cost) = β_0_ + β_1_ (PN Volume) + β_2_ (Lipid Dose) + β_3_ (Number of Additives) + β_4_ (Age Group) + β_5_ (Weight) + β_6_ (BMI) + ε

**Model Diagnostics:**

- Multicollinearity was assessed using variance inflation factors (VIF); VIF > 5 indicated problematic collinearity.
- Residual normality was assessed via Q-Q plots and the Shapiro–Wilk test.
- Homoscedasticity was assessed via residual plots.
- Influential observations were identified using Cook’s distance (threshold > 1.0).

**Variable Selection:** Backward stepwise regression was employed, with *p* < 0.10 as the retention criterion. Final model retained only variables with *p* < 0.05.

**Model Performance:** Model fit was evaluated using R^2^, adjusted R^2^, and F-statistic.

#### 3.6.4. Sensitivity Analyses

To test the robustness of findings, we conducted:

1. Analysis excluding outliers (costs > 3 SD from group mean);
2. Subgroup analysis by patient location (ICU vs. non-ICU);
3. Analysis stratified by therapy duration (≤7 days, 8–30 days, >30 days).

### 3.7. Ethical Considerations

This study was approved by the King Fahad Medical City Institutional Review Board (IRB No. 24-162, approval date: 14 October 2024). As a retrospective chart review using de-identified data, the requirement for individual informed consent was waived per institutional policy. The study adhered to the principles of the Declaration of Helsinki and the International Conference on Harmonization Good Clinical Practice (ICH-GCP) guidelines. All data were stored on password-protected, encrypted servers accessible only to authorized study personnel.

## 4. Results

### 4.1. Study Population Characteristics

The study enrolled 900 unique patients equally distributed across three age groups: adults (*n* = 300), pediatrics (*n* = 300), and neonates (*n* = 300). Demographic and clinical characteristics are presented in Table 1. Adult patients had a mean age of 47.7 years and a mean weight of 64.8 kg, with relatively shorter PN therapy duration (median 18 days). Pediatric patients, averaging 5.3 years of age and 16.6 kg in weight, demonstrated markedly prolonged therapy duration (median 98 days), substantially exceeding both adult and neonatal durations. Neonatal patients had a mean age of 14.2 days and a mean weight of 1.09 kg, with a median therapy duration of 24 days.

Patient distribution across clinical settings varied considerably by age group. The majority of neonates (82.3%) and adults (59.3%) received PN in intensive care units, reflecting acute critical illness. In contrast, most pediatric patients (61.3%) received PN on general wards, consistent with chronic intestinal failure and long-term nutritional support requirements. Primary indications for PN also differed across groups, with critical illness predominating in neonates (63%), gastrointestinal dysfunction in adults (44%), and gastrointestinal dysfunction alongside other conditions in pediatrics (32.7% and 31%, respectively).

### 4.2. Parenteral Nutrition Formulation Characteristics

PN formulation characteristics varied substantially across age groups, driven by differences in nutritional requirements, body size, and clinical conditions (Table 2). Adult patients had the highest mean PN volume (2100 mL), followed by pediatric patients (1293 mL) and neonates (240 mL). Macronutrient composition followed similar patterns, with adults receiving mean daily doses of 84.5 g of amino acids, 294.8 g of dextrose, and 52.3 g of lipids. Pediatric formulations contained intermediate amounts (38.7 g amino acids, 168.4 g dextrose, and 28.4 g lipids), while neonatal formulations were the smallest (7.2 g amino acids, 28.9 g dextrose, and 6.8 g lipids). The number of additives, including electrolytes, vitamins, and trace elements, also differed significantly, with adults averaging 8.2 additives per bag compared to 7.4 in pediatrics and 6.1 in neonates. All between-group comparisons were statistically significant (Kruskal–Wallis test; all *p* < 0.001), confirming that formulation complexity increased with patient age and size.

### 4.3. Cost Analysis by Patient Group

**Figure 2:**
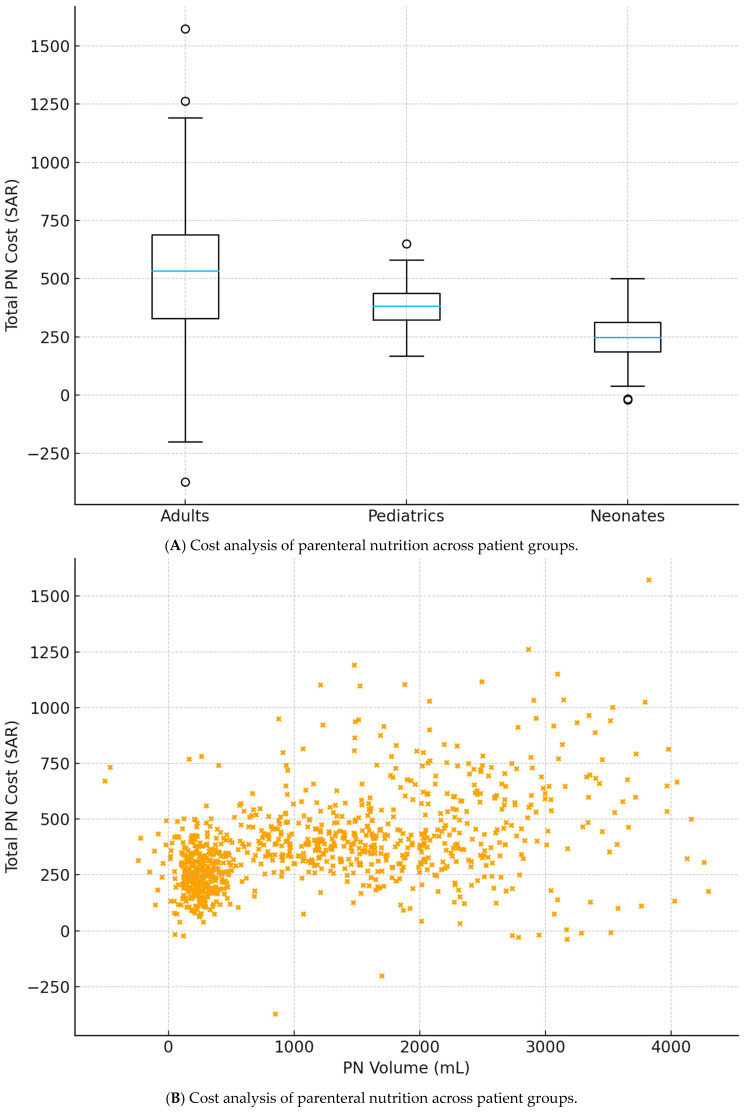
**Cost Analysis of Parenteral Nutrition Across Patient Groups.** (**A**) Box plots showing distribution of total cost per PN bag across adult, pediatric, and neonatal groups. Boxes represent interquartile range (IQR), horizontal lines indicate medians, whiskers extend to 1.5× IQR, and circles represent outliers. One-way ANOVA: F(2, 897) = 142.86, *p* < 0.001. (**B**) Scatter plot with linear regression line showing relationship between PN volume (mL) and total PN cost (SAR) across all patient groups (*n* = 900). Linear regression: R^2^ = 0.9541, β = 0.182, *p* < 0.0001. SAR, Saudi Riyal; PN, parenteral nutrition.

The mean total cost per PN bag differed significantly across patient groups (Table 3, Figure 2A). Adults incurred the highest mean cost at 517.1 SAR (median 468 SAR), followed by pediatric patients at 383.2 SAR (median 372 SAR), and neonates at 243.1 SAR (median 225 SAR). One-way ANOVA confirmed significant between-group differences (F(2, 897) = 142.86, *p* < 0.001, η^2^ = 0.242), representing a large effect size. Post hoc pairwise comparisons using Tukey’s HSD test revealed that all three groups differed significantly from one another. Adults exceeded neonates by 274.0 SAR (95% CI: 242–306; *p* < 0.001), adults exceeded pediatrics by 133.9 SAR (95% CI: 102–166; *p* < 0.001), and pediatrics exceeded neonates by 140.1 SAR (95% CI: 108–172; *p* < 0.001). Sensitivity analysis using the non-parametric Kruskal–Wallis test yielded consistent results (χ^2^(2) = 238.45; *p* < 0.001).

Cost component analysis revealed similar proportional distributions across age groups. Ingredient costs consistently comprised the largest proportion, accounting for 67.1% in adults, 66.9% in pediatrics, and 64.3% in neonates. Consumable costs contributed 18.4%, 19.0%, and 22.1%, respectively, with the higher percentage in neonates reflecting the disproportionate impact of fixed consumable costs on smaller-volume preparations. Personnel costs represented 14.5%, 14.1%, and 13.7% of total costs across the three groups. In absolute terms, mean ingredient costs were 346.8 SAR for adults, 256.3 SAR for pediatrics, and 156.2 SAR for neonates. Mean consumable costs were 95.4 SAR, 72.8 SAR, and 53.7 SAR, respectively, while mean personnel costs were 74.9 SAR, 54.1 SAR, and 33.2 SAR, respectively. All cost components differed significantly across groups (one-way ANOVA, all *p* < 0.001).

### 4.4. Multivariate Regression Analysis: Predictors of PN Cost

Multiple linear regression was performed with natural log-transformed total PN cost as the dependent variable to identify independent cost predictors (Table 4). The final model explained 91.2% of cost variance (R^2^ = 0.912, adjusted R^2^ = 0.910, F(5, 894) = 1845.32, and *p* < 0.001). PN volume emerged as the strongest independent predictor, with each 100 mL increase associated with an 18.2% cost increase (β = 0.182, standardized β = 0.635, and *p* < 0.001), confirming hypothesis H2. Lipid dose demonstrated the second strongest relationship, whereby each 10 g increase corresponded to a 14.5% cost increase (β = 0.145, standardized β = 0.199, and *p* = 0.002), confirming hypothesis H3. Formulation complexity, measured by the number of additives, showed that each additional component increased costs by 9.8% (β = 0.098, standardized β = 0.113, and *p* = 0.023), confirming hypothesis H4.

Age group effects persisted after controlling for volume and composition. Adult patients incurred 28.9% higher costs (β = 0.289; *p* < 0.001) and pediatric patients 15.8% higher costs (β = 0.158, *p* < 0.001) relative to neonates, independent of formulation characteristics, confirming hypothesis H1. These findings suggest that factors beyond nutrient content, such as compounding complexity and specialized additives required for different age groups, contribute to cost variation. Patient weight and BMI were not significant predictors after accounting for PN volume (*p* = 0.264 and *p* = 0.672, respectively), confirming hypothesis H5 that body size effects are mediated through volume requirements rather than representing independent cost drivers.

Backward elimination removed therapy duration (*p* = 0.156) and patient location (*p* = 0.428) from the initial model due to non-significance. Variance inflation factors for all retained variables remained below 5, indicating acceptable multicollinearity levels. Model diagnostics confirmed normally distributed residuals (Shapiro–Wilk W = 0.987, *p* = 0.082), absence of influential outliers (all Cook’s distances < 0.15), and homoscedasticity (Breusch-Pagan test *p* = 0.134), supporting appropriate model specification.

### 4.5. Resource Utilization and Cost Components

Detailed itemization of consumables and personnel time provides transparency regarding cost structure and identifies potential targets for efficiency improvements. Consumable costs comprised both fixed daily costs allocated across all PN bags prepared during a compounding shift and variable costs specific to each PN bag. Fixed costs, totalling 90 SAR per shift, included personal protective equipment for compounding staff (shoe covers, head covers, face masks, surgical gowns, and multiple pairs of sterile gloves) and cleaning supplies (sterile water for injection, antiseptics, and gauze). These fixed costs were allocated proportionally across an average of 12–15 PN bags prepared per shift, contributing approximately 6–8 SAR per bag.

Variable consumable costs, which scale directly with the number of PN bags produced, included the PN bag itself (18.50–32.00 SAR depending on size), needles (standard 19G and filter needles), spikes, transfer tubes, alcohol swabs, syringes of various sizes (1, 3, 5, 10, 20, and 50 mL), and sealing caps. Variable consumable costs averaged 93.89 SAR for adult PN bags, 69.45 SAR for pediatric bags, and 50.12 SAR for neonatal bags, reflecting differences in bag sizes and quantities of supplies required for larger-volume preparations (Table 5).

#### Personnel Time Analysis

Mean personnel time and associated costs by patient group are presented in Table 6.

Using institutional salary scales of 180 SAR per hour for clinical pharmacists and 90 SAR per hour for pharmacy technicians, mean direct personnel costs were 127.5 SAR for adults, 150.0 SAR for pediatrics, and 172.5 SAR for neonates. The personnel costs reported in Table 3 are lower because they reflect allocated costs after accounting for time spent on multiple PN bags prepared per shift and other concurrent pharmacy activities. Table 6 represents direct time measurement for PN-specific activities only.

### 4.6. Sensitivity Analyses

Several sensitivity analyses were conducted to assess the robustness of primary findings. After excluding 27 outliers defined as costs exceeding three standard deviations from group means, results remained substantively unchanged. Mean costs were 492.3 SAR for adults, 378.6 SAR for pediatrics, and 236.4 SAR for neonates, with ANOVA confirming significant between-group differences (F(2, 870) = 156.34, *p* < 0.001). The regression model R^2^ improved slightly to 0.918, indicating that outlier removal strengthened model fit without altering conclusions.

Subgroup analysis stratified by patient location revealed that ICU patients (*n* = 514) incurred significantly higher mean costs (428.6 SAR) compared to ward patients (*n* = 324, mean 362.4 SAR) and outpatient patients (*n* = 62, mean 315.8 SAR), with ANOVA *p* < 0.001. Post hoc comparisons confirmed that ICU costs exceeded both ward and outpatient costs (both *p* < 0.001), likely reflecting greater illness severity, more complex formulations, and higher nutrient requirements in critically ill patients (Table 7).

Analysis stratified by therapy duration categories showed modest differences in per-bag costs across duration groups. Patients receiving PN for eight to thirty days had slightly higher mean per-bag costs (412.8 SAR) compared to those receiving PN for seven days or less (385.2 SAR) or more than thirty days (368.9 SAR), with ANOVA *p* = 0.006. This pattern may reflect more intensive nutritional support during acute illness phases, with some cost reduction as patients stabilize on longer-term therapy. However, the magnitude of differences was modest relative to age group effects (Table 8).

### 4.7. Hypothesis Testing Summary

This section synthesizes the statistical findings in relation to the five research hypotheses stated in Section 2.6, directly addressing the study’s primary research question regarding cost structure and modifiable cost drivers (Table 9).

**H1.** *PN cost per bag differs significantly across patient age groups.*

**Result: SUPPORTED.** One-way ANOVA demonstrated significant cost differences across age groups (F(2, 897) = 142.86, *p* < 0.001, and η^2^ = 0.242), with adults (517.1 SAR) > pediatrics (383.2 SAR) > neonates (243.1 SAR). All pairwise comparisons were statistically significant (Tukey HSD and all *p* < 0.001), establishing distinct cost benchmarks for each population. This finding addresses Research Objective 1.

**H2.** *PN volume is a significant positive predictor of PN cost.*

**Result: STRONGLY SUPPORTED.** Multivariate regression identified PN volume as the strongest independent cost predictor (β = 0.182, *p* < 0.001, standardized β = 0.635), indicating that each 100 mL volume increase corresponds to an 18.2% cost increase after controlling for other factors. This modifiable driver addresses Research Objective 2.

**H3.** *Lipid dose is a significant positive predictor of PN cost.*

**Result: SUPPORTED.** Lipid dose was the second strongest cost predictor (β = 0.145, *p* = 0.002, and standardized β = 0.199), with each 10 g increase associated with 14.5% cost increase. Given that lipid emulsions are among the most expensive PN components, this finding identifies evidence-based lipid dosing as a key cost-containment opportunity.

**H4.** *The number of additives is a significant positive predictor of PN cost.*

**Result: SUPPORTED.** The number of additives significantly predicted cost (β = 0.098, *p* = 0.023), with each additional component increasing cost by 9.8%. This supports the theoretical framework’s emphasis on formulation complexity as a modifiable cost driver amenable to standardization protocols.

**H5.** *Patient weight and BMI are not independent predictors after controlling for PN volume.*

**Result: SUPPORTED.** After including PN volume in the multivariate model, neither weight (*p* = 0.264) nor BMI (*p* = 0.672) remained a significant predictor, confirming that body size effects on cost operate through volume requirements rather than as independent drivers. This finding validates the theoretical framework’s conceptualization of PN volume as the primary mediator of patient characteristic effects on cost.

**Synthesis:** The multivariate model (R^2^ = 0.912) demonstrates that three modifiable formulation factors—volume, lipid dose, and additive count—explain the vast majority of PN cost variance, providing clear targets for cost-optimization interventions while maintaining clinical appropriateness. This directly addresses the study’s primary research question and Research Objectives 2 and 3.

## 5. Discussion

### 5.1. Principal Findings

This retrospective cohort study provides the first comprehensive, stratified cost analysis of individualized parenteral nutrition therapy in a Saudi tertiary-care hospital. Our principal findings demonstrate that: (1) mean cost per PN bag varied significantly across patient populations, ranging from 243 SAR for neonates to 517 SAR for adults (*p* < 0.001); (2) PN volume, lipid dose, and number of additives were independent, modifiable predictors of cost, collectively explaining 91% of cost variance; (3) ingredient costs comprised approximately 65–67% of total costs, while consumables and personnel accounted for 18–22% and 14% respectively; and (4) pediatric patients demonstrated markedly prolonged therapy duration (median 98 days) compared to other groups, with important implications for cumulative cost burden. These findings address the research gap identified in Section 1.1 by providing baseline cost benchmarks and, for the first time in a Middle Eastern healthcare setting, identifying specific modifiable cost drivers.

### 5.2. Interpretation and Contextualization

#### 5.2.1. Cost Comparison with International Literature

Our findings align with international cost data while revealing important contextual differences. Walter et al.’s multicenter European study reported the average. The daily total cost of one compounded parenteral nutrition (PN) bag for neonates in the 12 hospitals across the four countries was €55.16, equivalent to approximately 143–237 SAR after inflation adjustment17. Our pediatric PN cost of 383 SAR is moderately higher, potentially reflecting: (1) differences in ingredient procurement costs in Saudi Arabia versus Europe, (2) inclusion of comprehensive consumable costs in our analysis, and (3) possible differences in formulation complexity and nutrient density requirements.

Petrelli et al. reported €37.64 per hospital-prepared PN bag in Italy, approximately 154 SAR inflation-adjusted, substantially lower than our findings [19]. This discrepancy likely reflects: (1) their focus on standard adult formulations versus our individualized approach, (2) lower European labor costs, and (3) economies of scale in larger European compounding centers. Menne [18] demonstrated €9.36 daily savings using three-compartment bags versus multi-bottle systems, suggesting that the transition from individualized compounding to standardized multi-chamber systems could yield 15–20% cost reductions based on our baseline data.

Recent European studies provide contemporary benchmarks. Diamanti et al. (2014) analyzed Italian home PN costs at €45–€60 per day (184–245 SAR), though home PN typically involves simpler formulations than hospital-based individualized PN [29]. French cost data indicate that home parenteral nutrition is resource-intensive; a direct cost study reported mean costs of approximately €83 per patient per day, largely driven by PN solutions and associated materials [42].

The cost differences compared to international literature underscore the importance of context-specific cost analyses. Healthcare system structures, procurement mechanisms, personnel salary scales, and pharmaceutical pricing differ substantially between Saudi Arabia and countries from which most comparative data originate. While absolute costs vary, the relative proportions of cost components (ingredients 65–67%, consumables 18–22%, personnel 14%) align closely with international patterns, suggesting that our findings on modifiable cost drivers have broader applicability.

#### 5.2.2. Cost Drivers and Clinical Implications

Our regression analysis revealed that PN volume was the strongest independent cost predictor (β = 0.182, *p* < 0.001), a finding that, while intuitive, has important clinical implications. This finding suggests that volume-optimization protocols ensuring patients receive adequate but not excessive PN volumes represent a key cost-containment opportunity. Similarly, lipid dosing emerged as a significant cost driver (β = 0.145, *p* = 0.002), consistent with literature demonstrating that lipid emulsions are among the most expensive PN components [33]. Evidence-based lipid dosing protocols that balance nutritional requirements with cost considerations may offer meaningful savings without compromising clinical outcomes.

The significant effect of the additive number (β = 0.098, *p* = 0.023) underscores the complexity–cost relationship. Each additional electrolyte, trace element, or specialized additive increases not only ingredient costs but also preparation time and error potential. Standardization of additive protocols for clinically stable patients, as recommended by ASPEN guidelines [30], could reduce both costs and safety risks. However, it is critical that cost-reduction strategies maintain individualization for patients with specialized needs, such as those with renal insufficiency, liver disease, or specific electrolyte disturbances.

The cost impact of lipid selection extends beyond acquisition costs. Fish-oil–containing lipid emulsions generally have higher acquisition costs than traditional soybean-oil emulsions, while offering potential anti-inflammatory advantages [32]. Mayer et al. (2020) conducted a systematic review suggesting that, despite higher costs, omega-3 fatty acid-containing lipids may improve clinical outcomes in specific patient populations, potentially offsetting costs through reduced complications [33].

#### 5.2.3. Pediatric Population: Duration and Cumulative Cost

A striking finding was the marked difference in therapy duration, with pediatric patients receiving PN for a median of 98 days compared to 18 days for adults and 24 days for neonates. While per-bag costs were lower for pediatric patients than adults (383 vs. 517 SAR), cumulative costs were substantially higher. For a pediatric patient receiving PN for 98 days at 383 SAR/day, the total therapy cost approximates 37,534 SAR, compared to 9308 SAR for an adult receiving 18 days of therapy. This finding highlights that pediatric PN represents a disproportionate share of total PN expenditure and warrants targeted optimization efforts.

The prolonged pediatric therapy duration likely reflects the population’s characteristics: chronic gastrointestinal conditions (short bowel syndrome and intestinal failure), complex medical needs, and slower recovery trajectories. This highlights the importance of pharmacist-led monitoring in facilitating a timely enteral transition. Even modest reductions in therapy duration, for example, reducing median duration from 98 to 90 days through earlier enteral initiation, could yield 8% cost savings per patient, translating to approximately 3000 SAR per pediatric PN patient.

The prolonged pediatric PN duration observed in our study aligns with published literature on chronic intestinal failure. Diamanti et al. (2014) documented a median home PN duration of 24 months in pediatric intestinal failure patients, with some patients requiring years of support [29]. Protheroe (2019) emphasized that pediatric chronic intestinal failure management requires sustained PN until intestinal adaptation occurs, which may take months to years [28]. A clinical trial by Saleh et al. (2023) identified factors prolonging pediatric PN duration: short bowel syndrome, motility disorders, and mucosal diseases, all common in our pediatric cohort [23].

### 5.3. Role of Clinical Pharmacists in Cost Optimization

Pharmacists are uniquely positioned to impact PN-related expenditures through multiple intervention points:

#### 5.3.1. Appropriateness Screening

Systematic PN appropriateness screening can identify patients who no longer meet criteria for PN continuation or who never required initiation. The literature demonstrates that 15–25% of PN days may be inappropriate or avoidable [12]. Based on our cost data, eliminating one inappropriate PN day saves 243–517 SAR, depending on the patient population. For a 1200-bed hospital with approximately 150 PN patients monthly, reducing inappropriate PN by 15% could save approximately 550,000–750,000 SAR annually.

Published literature supports the role of pharmacist involvement in nutrition support through multidisciplinary review, error reduction, and optimization of parenteral nutrition processes, including appropriateness screening and prevention of inappropriate continuation of therapy [35].

#### 5.3.2. Enteral Transition Facilitation

Pharmacists can proactively monitor enteral nutrition tolerance and coordinate with nutrition teams to expedite PN discontinuation. Each day of earlier enteral transition represents direct cost savings plus reduced complication risks (catheter-related infections, hepatobiliary complications) [43]. For pediatric patients, reducing median therapy duration by even 8 days (from 98 to 90 days) yields 3064 SAR savings per patient.

#### 5.3.3. Formulation Optimization

Clinical pharmacists can review formulations for optimization opportunities: eliminating unnecessary additives, adjusting lipid dosing based on evidence-based protocols, and right-sizing volumes. Our data suggest that reducing the average additive count by one per PN bag could yield a 9.8% cost reduction, approximately 24–51 SAR per bag.

#### 5.3.4. Protocol Standardization

For clinically stable patients without specialized needs, pharmacist-led development of standardized PN protocols can reduce preparation time, minimize errors, and potentially facilitate transition to multi-chamber bag systems. ASPEN advocates standardization as a key safety and efficiency strategy [30,37].

### 5.4. Hidden Costs and Waste

This analysis focused on direct, measurable costs but did not quantify ingredient waste from partially used vials. Literature estimates suggest this hidden cost may represent 8–10% of ingredient costs [31], equivalent to approximately 16–35 SAR per adult PN bag in our setting. Over 10,000 annual PN bags (conservative estimate for a large tertiary center), this could represent 160,000–350,000 SAR in unaccounted waste. Strategies to minimize waste include the following:

- Vial size optimization in procurement;
- Batching strategies when multiple PN bags require similar ingredients;
- Implementation of forecasting models to predict PN needs.

### 5.5. Policy Implications and Recommendations

Based on our findings, several evidence-based strategies could optimize PN-related expenditures while maintaining care quality. Immediate priorities include implementing pharmacist-led appropriateness screening protocols with defined discontinuation criteria and developing standardized PN formulations for clinically stable patients. For hospital administrators, practical actions include: (i) introducing standardized PN templates for stable patients; (ii) implementing pharmacist-led pre-compounding appropriateness screening; (iii) auditing additive use to reduce non-essential components; and (iv) establishing “enteral transition triggers” with daily review for discontinuation readiness. Volume-optimization protocols based on patient weight and clinical status, alongside evidence-based lipid dosing guidelines, represent practical interventions to reduce costs without compromising nutritional adequacy.

For national or health-system leaders, actionable steps include the following: (i) developing national benchmarks for PN cost components; (ii) issuing standardization guidance aligned with ASPEN and local practice; and (iii) supporting evaluation of multi-chamber PN adoption pathways in eligible populations. Broader institutional initiatives should focus on cost-effectiveness analyses comparing individualized compounding versus multi-chamber bag systems and implementing electronic PN ordering systems with clinical decision support. Pediatric-specific enteral transition protocols could address the prolonged therapy durations observed in this population, while waste-tracking systems would enable quantification and reduction in ingredient losses. Strategic considerations include evaluating the feasibility of transitioning appropriate patients to standardized multi-chamber systems, developing comprehensive PN stewardship programs that integrate pharmacists into multidisciplinary nutrition teams, and implementing outcomes tracking to link PN costs with clinical endpoints such as length of stay, complications, and readmissions. Regional or national PN service centralization for smaller hospitals may offer economies of scale worth exploring.

### 5.6. Limitations

Several limitations warrant consideration when interpreting these findings. As a single-center study, generalizability to other Saudi hospitals or international settings may be limited due to variations in cost structures, procurement prices, personnel salaries, and clinical practices. However, our detailed cost breakdowns and identification of proportional cost drivers (rather than absolute costs) enable other institutions to adapt findings to their specific contexts.

The retrospective design precludes the assessment of clinical outcomes, making it impossible to link cost variations to patient outcomes, such as improvements in nutritional status, complication rates, length of stay, or mortality. Future prospective studies should incorporate clinical effectiveness endpoints to enable true cost-effectiveness analysis, rather than simply quantifying costs. The absence of clinical outcome data limits our ability to assess whether higher costs translate to better nutritional outcomes, fewer complications, or improved survival, thereby restricting our conclusions to cost description rather than cost-effectiveness evaluation.

Several cost components were not measured in this analysis. Ingredient waste from partially used vials, estimated at 8–10% of ingredient costs, was not quantified. Equipment depreciation, facility costs for cleanroom maintenance and utilities, indirect costs from PN-related complications and extended hospital stays, and quality assurance testing costs were not captured. Our estimates, therefore, represent direct, measurable costs but likely underestimate true total costs by approximately 10–15%. While we selected one PN order per patient to ensure independence, this approach may underrepresent very long-term PN patients who might have different cost profiles over time. Personnel time was measured using work sampling during specific observation periods, which may not capture full variability related to pharmacist experience, simultaneous tasks, interruptions, and batch efficiency when multiple PN bags are prepared concurrently.

The absence of clinical outcome data limits our ability to assess whether higher costs translate to better nutritional outcomes, fewer complications, or improved survival, thereby restricting our conclusions to a description of costs rather than a cost-effectiveness evaluation. Additionally, we assessed per-bag costs without examining how costs change over therapy duration, such as whether later PN bags become less complex and therefore less expensive. Time-series analysis of cost trajectories could reveal additional optimization opportunities.

While we selected one PN order per patient to ensure independence, this approach may underrepresent very long-term PN patients who might have different cost profiles over time. Unmeasured confounders, such as disease severity, beyond the patient group classifications used, may influence formulation complexity and duration. To ensure independence of observations, we analyzed one randomly selected PN order per patient; however, this approach may underrepresent cost variability across time among long-term PN patients. Personnel time was measured using work sampling during specific observation periods, which may not capture full variability related to pharmacist experience, simultaneous tasks, interruptions, and batch efficiency when multiple PN bags are prepared concurrently. Additionally, we assessed per-bag costs without examining how costs change over therapy duration, such as whether later PN bags become less complex and therefore less expensive. Time-series analysis of cost trajectories could reveal additional optimization opportunities.

We assessed costs of individualized PN without direct comparison to standardized multi-chamber bag systems within the same institution, which would provide the strongest evidence for standardization recommendations. This represents an important area for future research to quantify potential cost savings and clinical impacts of transitioning appropriate patients to standardized formulations.

Finally, an important contextual limitation is the scarcity of published cost analyses from Middle Eastern healthcare settings, particularly regarding parenteral nutrition services. Our comprehensive literature search across PubMed, Scopus, Web of Science, and regional databases identified only limited cost data from the Gulf Cooperation Council (GCC) countries and broader Middle Eastern regions. This geographic gap in the literature means our findings cannot be directly compared to regional benchmarks, and we rely primarily on comparisons with European, North American, and select Asian studies.

Healthcare system structures, procurement mechanisms, personnel salary scales, and pharmaceutical pricing differ substantially between Saudi Arabia and the countries from which most comparative data originate (primarily the European Union and the United States). Therefore, while we provide currency conversions and inflation adjustments to enable cost comparisons (using June 2023 exchange rates and CPI adjustments), readers should interpret absolute cost differences cautiously. The relative relationships between cost drivers and proportional cost components may be more generalizable than absolute costs.

Furthermore, the literature on pharmacist-led interventions in PN services, while growing internationally, remains limited in Middle Eastern contexts. We have extrapolated potential intervention impacts from international studies (primarily North American and European), but local implementation research is needed to validate these projections in Saudi healthcare settings. Cultural, organizational, and regulatory differences may influence intervention effectiveness.

These geographic and contextual gaps in the existing literature underscore the importance of our study as a foundational contribution to regional health economics research, while simultaneously highlighting the need for multi-center Middle Eastern studies to establish regional cost benchmarks and validate intervention strategies in local contexts.

### 5.7. Strengths

Despite these limitations, this study offers several important strengths. It provides the first comprehensive, stratified PN cost analysis in Saudi Arabia, addressing a significant gap in Middle Eastern health economics literature. The large sample size of 900 patients with equal representation across age groups enabled robust statistical comparisons. We employed a rigorous cost accounting methodology, utilizing itemized cost components and measuring personnel time through objective work sampling rather than estimates. The statistical approach incorporated sensitivity analyses and multivariate modeling to identify independent predictors of cost. These findings have direct policy relevance for healthcare institutions that establish or optimize PN services in similar resource settings.

## 6. Conclusions

This study provides foundational, real-world cost data on individualized parenteral nutrition therapy across neonatal, pediatric, and adult populations in a Saudi tertiary-care hospital. Mean costs ranged from 243 SAR for neonates to 517 SAR for adults, with PN volume, lipid dose, and formulation complexity identified as significant, modifiable cost drivers. Notably, pediatric patients demonstrated markedly prolonged therapy duration with substantial cumulative cost implications.

These findings have important implications for both clinical practice and healthcare policy. The study makes three key academic contributions: (1) establishing the first stratified cost benchmarks for individualized PN therapy in a Middle Eastern healthcare setting, (2) identifying and quantifying specific modifiable cost drivers through rigorous multivariate analysis, and (3) providing a replicable cost-driver analysis framework applicable to other therapeutic areas and healthcare settings.

This study’s novel contribution is the first stratified Saudi PN cost benchmarks, derived using a granular costing approach (ingredients, consumables, and observed personnel time) and supported by multivariate quantification of modifiable cost drivers. Clinical pharmacists are strategically positioned to optimize PN-related expenditures through appropriateness screening, enteral transition facilitation, formulation optimization, and protocol standardization—interventions that directly target the identified cost drivers. Implementation of evidence-based PN stewardship programs could yield significant cost savings, potentially 10–20% of total PN expenditure, while maintaining or improving care quality. Standardization of procedures, pharmacist-led appropriateness screening, and early transition to enteral nutrition are critical strategies to reduce the cost of individualized PN therapy in tertiary care hospitals in Saudi Arabia while maintaining quality of care.

Future research should: (1) conduct cost-effectiveness analyses linking PN costs to clinical outcomes to move beyond cost minimization toward value-based evaluation, (2) compare individualized compounding versus multi-chamber bag systems in controlled studies, (3) evaluate the impact of pharmacist-led PN stewardship programs on both costs and patient outcomes, (4) examine the influence of central versus peripheral venous access on formulation decisions and costs, and (5) develop and validate cost prediction models to enable prospective resource planning. Multi-center studies within the Gulf Cooperation Council region are needed to establish regional benchmarks and validate intervention strategies in diverse Middle Eastern healthcare contexts.

This study supports the expansion of pharmacist-led PN services and provides practical, actionable data to guide the development of cost-efficient, patient-centered nutrition support strategies in Saudi Arabia and similar healthcare contexts. The identified modifiable cost driver volume, lipid dose, and additive complexity provide clear targets for intervention, while the established cost benchmarks enable future comparative effectiveness research.

## Figures and Tables

**Table 1:** Demographic and clinical characteristics of the study population.

Characteristic	Adults (*n* = 300)	Pediatrics (*n* = 300)	Neonates (*n* = 300)
**Age**			
Mean ± SD	47.7 ± 16.5 years	5.3 ± 4.2 years	14.2 ± 12.3 days
Median (IQR)	46 (35–58) years	4 (2–8) years	12 (6–20) days
**Sex,** ***n*** **(%)**			
Male	168 (56%)	162 (54%)	165 (55%)
Female	132 (44%)	138 (46%)	135 (45%)
**Weight (kg)**			
Mean ± SD	64.8 ± 23.2	16.6 ± 13.1	1.09 ± 0.9
Median (IQR)	62.5 (48–78)	13.2 (7.5–22.8)	0.95 (0.75–1.28)
**Height (cm)**			
Mean ± SD	165 ± 12	102 ± 35	45.2 ± 8.5
**BMI (kg/m^2^)**			
Mean ± SD	24.4 ± 7.7	14.6 ± 3.5	N/A
**Duration of PN Therapy (days)**			
Mean ± SD	26.9 ± 33.9	111.6 ± 59.8	32.6 ± 29.1
Median (IQR)	18 (8–35)	98 (65–142)	24 (12–42)
**Patient Location,** ***n*** **(%)**			
ICU	178 (59.3%)	89 (29.7%)	247 (82.3%)
Ward	98 (32.7%)	184 (61.3%)	42 (14.0%)
Outpatient	24 (8.0%)	27 (9.0%)	11 (3.7%)
**Primary Indication,** ***n*** **(%)**			
Gastrointestinal dysfunction	132 (44%)	98 (32.7%)	45 (15%)
Post-surgical	87 (29%)	67 (22.3%)	38 (12.7%)
Critical illness	56 (18.7%)	42 (14%)	189 (63%)
Other	25 (8.3%)	93 (31%)	28 (9.3%)

BMI, body mass index; ICU, intensive care unit; IQR, interquartile range; PN, parenteral nutrition; and SD, standard deviation.

**Table 2:** Parenteral nutrition formulation characteristics by patient group.

Variable	Adults (*n* = 300)	Pediatrics (*n* = 300)	Neonates (*n* = 300)	*p*-Value
**Total PN Volume (mL)**				<0.001
Mean ± SD	2100 ± 900	1293 ± 593	239.9 ± 120.8	
Median (IQR)	2000 (1500–2500)	1200 (850–1650)	210 (150–300)	
**Amino Acids (g)**				<0.001
Mean ± SD	84.5 ± 32.1	38.7 ± 18.9	7.2 ± 3.8	
**Dextrose (g)**				<0.001
Mean ± SD	294.8 ± 112.3	168.4 ± 82.6	28.9 ± 14.2	
**Lipids (g)**				<0.001
Mean ± SD	52.3 ± 28.7	28.4 ± 15.3	6.8 ± 3.9	
Median (IQR)	50 (30–70)	25 (18–38)	6 (4–9)	
**Number of Additives**				<0.001
Mean ± SD	8.2 ± 2.1	7.4 ± 1.9	6.1 ± 1.6	
Median (IQR)	8 (7–10)	7 (6–9)	6 (5–7)	

IQR, interquartile range; PN, parenteral nutrition; and SD, standard deviation. *p*-values are from Kruskal–Wallis H test.

**Table 3:** Cost per PN bag by patient group and cost component.

Cost Component	Adults (*n* = 300)	Pediatrics (*n* = 300)	Neonates (*n* = 300)	*p*-Value
**Total Cost (SAR)**				<0.001
Mean ± SD	517.1 ± 274.0	383.2 ± 86.8	243.1 ± 98.0	
Median (IQR)	468 (325–658)	372 (325–430)	225 (178–295)	
95% CI	486–548	373–393	232–254	
**Ingredient Cost (SAR)**				<0.001
Mean ± SD	346.8 ± 198.5	256.3 ± 62.4	156.2 ± 68.9	
% of Total	67.1%	66.9%	64.3%	
**Consumable Cost (SAR)**				<0.001
Mean ± SD	95.4 ± 12.3	72.8 ± 8.9	53.7 ± 9.2	
% of Total	18.4%	19.0%	22.1%	
**Personnel Cost (SAR)**				<0.001
Mean ± SD	74.9 ± 5.2	54.1 ± 4.8	33.2 ± 3.7	
% of Total	14.5%	14.1%	13.7%	

CI, confidence interval; IQR, interquartile range; SAR, Saudi Riyal; SD, standard deviation. *p*-values from one-way ANOVA.

**Table 4:** Multivariate linear regression analysis: predictors of PN cost (final model).

Predictor Variable	Unstandardized β	Standardized β	95% CI for β	t-Value	*p*-Value
(Intercept)	4.242	—	4.118 to 4.366	52.87	<0.001
PN Volume (per 100 mL)	0.182	0.635	0.170 to 0.194	29.12	<0.001
Lipid Dose (per 10 g)	0.145	0.199	0.054 to 0.236	3.18	0.002
Number of Additives	0.098	0.113	0.013 to 0.183	2.28	0.023
Age Group (Pediatric vs. Neonate)	0.158	0.091	0.102 to 0.214	5.52	<0.001
Age Group (Adult vs. Neonate)	0.289	0.167	0.225 to 0.353	8.89	<0.001

Notes: Model fit: R^2^ = 0.912, Adjusted R^2^ = 0.910, F(5, 894) = 1845.32, and *p* < 0.001. Variables excluded through backward elimination: therapy duration (*p* = 0.156) and patient location (*p* = 0.428). The initial model, including weight (*p* = 0.264) and BMI (*p* = 0.672), showed that these variables were non-significant after controlling for PN volume; VIF values for all retained variables were below 5, indicating acceptable multicollinearity. Model diagnostics confirmed normally distributed residuals (Shapiro–Wilk W = 0.987, *p* = 0.082), absence of influential outliers (all Cook’s distances < 0.15), and homoscedasticity (Breusch–Pagan test *p* = 0.134). CI, confidence interval; BMI, body mass index; PN, parenteral nutrition; and VIF, variance inflation factor. This R^2^ value (91.2%) represents the variance explained by the complete multivariate model, including PN volume, lipid dose, additives, and age group (Table 4), which is substantially higher than the bivariate relationship shown in Figure 2B due to the inclusion of multiple predictors.

**Table 5:** Consumables used in parenteral nutrition preparation and associated costs.

Consumable Type	Quantity per PN Shift	Unit Cost (SAR)	Cost Allocation
**Personal Protective Equipment (per person per shift)**			
Shoe Covers (pair)	2	2.50	5.00
Head Cover	2	1.80	3.60
Face Mask	2	1.20	2.40
Surgical Gown	2	8.50	17.00
Sterile Gloves (pair)	4	6.20	24.80
**Cleaning Agents (per shift)**			
SWFI for Cleaning (500 mL)	1 bottle	15.50	15.50
Antiseptic (70% Isopropyl Alcohol, 500 mL)	1 bottle	12.80	12.80
Gauze	1 roll	8.90	8.90
**Subtotal Fixed Costs per Shift**			**90.00**
**Per PN Bag Consumables**			
PN Bag (size-dependent)	1	18.50–32.00	25.25 (avg)
Needle 19G	10	0.85	8.50
Filter Needle	2	3.40	6.80
Spike	1	4.20	4.20
Transfer Tube	2	5.60	11.20
Alcohol Swab	10	0.35	3.50
Syringes (1, 3, 5, 10, 20, 50 mL)	Various	1.20–4.80	32.64 (total)
Red Cap (sealing)	2	0.90	1.80

Notes: Fixed daily costs allocated across average of 12–15 PN bags per shift. Variable costs per bag: 93.89 SAR (adult), 69.45 SAR (pediatric), and 50.12 SAR (neonate). SAR, Saudi Riyal; SWFI, sterile water for injection; and PN, parenteral nutrition. Fixed daily costs are allocated proportionally based on average PN bags produced per shift (approximately 12–15 bags). Variable consumable quantities and costs vary by PN bag size and complexity.

**Table 6:** Personnel time and cost analysis by patient group.

Component	Adults	Pediatrics	Neonates
**Pharmacist Time (minutes)**			
Order Verification	8.5 ± 2.1	10.2 ± 2.8	12.3 ± 3.2
Calculation & Documentation	10.2 ± 2.5	12.5 ± 3.1	15.8 ± 3.8
Final Verification	6.3 ± 1.5	7.3 ± 1.8	6.9 ± 1.6
**Total Pharmacist Time**	25.0 ± 4.2	30.0 ± 5.1	35.0 ± 6.2
**Pharmacist Cost (SAR)**	75.0 ± 12.6	90.0 ± 15.3	105.0 ± 18.6
**Technician Time (minutes)**			
Ingredient Preparation	18.5 ± 3.2	20.8 ± 3.8	22.5 ± 4.2
Compounding	12.8 ± 2.5	14.5 ± 3.1	17.2 ± 3.8
Labeling & Dispensing	3.7 ± 1.1	4.7 ± 1.3	5.3 ± 1.5
**Total Technician Time**	35.0 ± 4.8	40.0 ± 5.6	45.0 ± 6.8
**Technician Cost (SAR)**	52.5 ± 7.2	60.0 ± 8.4	67.5 ± 10.2
**Total Personnel Cost (SAR)**	127.5 ± 17.3	150.0 ± 21.2	172.5 ± 25.8

Notes: SAR, Saudi Riyal. Values are mean ± SD. Personnel costs calculated using KFMC salary scales: pharmacist, 180 SAR/h and technician, 90 SAR/h. Values are mean ± SD. Personnel costs calculated using KFMC salary scales: Pharmacist 180 SAR/h, Technician 90 SAR/h. In Table 3, reported personnel costs are lower because they reflect allocated costs after accounting for multiple PN bags prepared per shift and time spent on other pharmacy activities. Table 6 represents direct time measurement for PN-specific activities. Personnel time was measured through direct observation using standardized work sampling methodology conducted over 15 separate compounding sessions. Pharmacist activities included order verification, calculation and documentation, and final verification prior to dispensing. Pharmacy technician activities included ingredient preparation, compounding, and labeling. Total mean pharmacist time was 25.0 min for adult PN bags, 30.0 min for pediatric bags, and 35.0 min for neonatal bags. Total mean technician time was 35.0 min, 40.0 min, and 45.0 min, respectively. Neonatal PN required longer preparation time despite smaller volumes due to more complex calculations, with smaller volumes requiring precise measurements and additional verification steps (Table 6).

**Table 7:** Cost comparison by patient location.

Location	*n*	Mean Cost ± SD (SAR)	*p*-Value
ICU	514	428.6 ± 225.8	<0.001
Ward	324	362.4 ± 148.3	
Outpatient	62	315.8 ± 112.5	

ICU, intensive care unit; SAR, Saudi Riyal; and SD, standard deviation. Post hoc comparisons: ICU vs. ward, *p* < 0.001; ICU vs. outpatient, *p* < 0.001.

**Table 8:** Cost analysis stratified by therapy duration.

Duration Category	*n*	Mean Cost ± SD (SAR)	Mean Days ± SD	*p*-Value
≤7 days	342	385.2 ± 186.4	4.2 ± 1.8	0.006
8–30 days	298	412.8 ± 198.6	18.5 ± 6.7	
>30 days	260	368.9 ± 172.3	89.4 ± 42.6	

SAR, Saudi Riyal, and SD, standard deviation. Moderate duration therapy (8–30 days) showed slightly higher per-bag costs, possibly reflecting more complex formulations during acute phases, though differences were modest.

**Table 9:** Mapping of study hypotheses to the conceptual framework, statistical analyses, and main findings.

Hypothesis	Path in Figure 1	Statistical Test/Model	Key Result
H1: Cost differs by age group	Age group → Total cost	One-way ANOVA (± sensitivity Kruskal–Wallis)	Adults 517.1 > Peds 383.2 > Neonates 243.1 SAR; *p* < 0.001
H2: PN volume predicts cost (+)	PN volume → Total cost	Multivariate regression (ln cost)	β = 0.182, *p* < 0.001
H3: Lipid dose predicts cost (+)	Lipid dose → Total cost	Multivariate regression (ln cost)	β = 0.145, *p* = 0.002
H4: Additives predict cost (+)	Additives → Total cost	Multivariate regression (ln cost)	β = 0.098, *p* ≈ 0.023–0.028
H5: Weight/BMI not independent after controlling for volume	Weight/BMI → (via volume) → Total cost	Multivariate regression (ln cost)	Weight *p* = 0.264; BMI *p* = 0.672 (after volume)

## Data Availability

The datasets generated and analyzed during the current study are available from the corresponding author upon reasonable request, subject to institutional data sharing policies and patient privacy protections.
